# Management of Blunt Sternal Fractures in a Community-Based Hospital

**DOI:** 10.1155/2023/8896989

**Published:** 2023-03-13

**Authors:** Fathima T. Kunhivalappil, Taleb M. Almansoori, Muhamed Salim AbdulRahman, Mohamed A. Hefny, Nirmin A. Mansour, Taoufik Zoubeidi, Moien A. B. Khan, Ashraf F. Hefny

**Affiliations:** ^1^Department of Radiology, Al Rahba Hospital, Abu Dhabi, UAE; ^2^Department of Radiology, CMHS, UAEU, Al Ain, UAE; ^3^Department of Surgery, Al Rahba Hospital, Abu Dhabi, UAE; ^4^Department of Surgery, Faculty of Medicine, Ain Shams University, Cairo, Egypt; ^5^Ambulatory Health Services, SEHA, Abu Dhabi, UAE; ^6^Department of Statistics, United Arab Emirates University, Al Ain, UAE; ^7^Department of Family Medicine, College of Medicine and Health Sciences, UAE University, Al Ain, UAE; ^8^Department of Surgery, College of Medicine and Health Sciences, UAE University, Al Ain, UAE

## Abstract

**Background:**

Sternal fractures are not commonly observed in patients with blunt trauma. The routine use of computed tomography (CT) in the evaluation of chest trauma helps identify these fractures. We studied the incidence, injury mechanism, management, and outcome of sternal fractures in patients with blunt trauma treated at our community-based hospital.

**Methods:**

We retrospectively reviewed the chest CT scans of all patients with blunt trauma who were presented to our community-based hospital from October 2010 to March 2019. The study variables included age at the time of injury, sex, mechanism of injury, type, and site of fracture, associated injuries, Glasgow Coma Scale, Injury Severity Score, need for intensive care unit admission, hospital stay, and long-term outcome.

**Results:**

In total, 5632 patients with blunt trauma presented to our hospital during the study period, and chest CT scan was performed for 2578 patients. Sternal fractures were diagnosed in 63 patients. The primary mechanism of injury was a motor vehicle collision. The most common site of fracture was the body of the sternum (47 patients; 74.6%). Twenty (31.7%) patients had an isolated sternal fracture with no other injuries. Seven (11.1%) patients were discharged directly from the emergency department. Two patients died (overall mortality rate, 3.2%) and two experienced long-term disability.

**Conclusions:**

The incidence of sternal fractures in our patient population was similar to that reported by tertiary hospitals. Patients with a sternal fracture and normal cardiac enzyme levels and electrocardiogram may be safely discharged from the emergency department, provided there are no other major injuries.

## 1. Introduction

A fracture of the sternum is an uncommon injury in patients with blunt trauma. It occurs in about 3% to 8% of patients with blunt trauma [1]. Motor vehicle collisions (MVCs) are the most common mechanism of blunt sternal fracture. Injuries to the chest wall have increased with the widespread use of seatbelts [2, 3]. The routine use of a computed tomography (CT) scan in the evaluation of blunt chest trauma helps identify sternal fractures [4]. Isolated sternal fractures are primarily treated with conservative management; however, a sternal fracture alone may be an indicator for other life-threatening injuries [2]. Sternal fractures cause direct injury to adjacent structures including the heart, lungs, and pleura. Blunt cardiac injuries are the most dangerous as they frequently lead to arrhythmias, cardiac tamponade, and sudden death [5].

The diagnosis of cardiac injury is usually based on clinical presentation, an abnormal electrocardiogram (ECG), and elevated levels of cardiac enzymes, such as troponin, creatine kinase (CK), and creatine kinase MB (CK-MB) [2]. The morbidity and mortality of patients with sternal fractures are mainly associated with concomitant injuries to the head, neck, chest, spine, and abdomen [6]. This study is a retrospective review of the incidence, injury mechanism, management, and outcome of sternal fractures in patients with blunt trauma who were treated at our community-based hospital.

## 2. Materials and Methods

Al Rahba Hospital is an advanced community-based hospital with a capacity of 190 beds. Approximately 6000 patients with trauma present to the hospital each year with 650 admissions annually. The hospital is managed by Johns Hopkins Medicine International on behalf of the Abu Dhabi Health Services/Department of Health of Abu Dhabi. This project was approved by the Al Rahba Hospital Research and Ethics Committee (ARH/REC-090).

One senior radiologist retrospectively reviewed chest CT scans of all patients with blunt trauma who were presented to our hospital from October 2010 to March 2019. All patient files with CT scans that revealed sternal fractures were reviewed. The following information was collected from patients' records: age at the time of injury, sex, mechanism of injury, associated injuries, Glasgow Coma Scale (GCS), Injury Severity Score (ISS), need for intensive care unit (ICU) admission, hospital stay, and outcome. Further, the electrocardiogram (ECG) and levels of cardiac enzymes, such as troponin, CK, and CK-MB, were reviewed.

Axial CT images were viewed in the lung, mediastinal, and bone windows. Multiplanar reformats were used to evaluate the sternum in the coronal and sagittal planes. Three-dimensional (3D) images of the sternum were reviewed in all patients. All CT scans were performed using a General Electric 64-slice Light Speed Volume scanner (GE 64-slice Light Speed VCT).

The CT scan criteria for the diagnosis of a sternal fracture included cortical disruption with or without displacement. The sternum was divided into five parts: the manubrium sterni, the upper body, the middle body, the lower body, and the xiphisternum to localize the fracture within the sternum. Isolated sternal fractures were defined as the only injury without any additional skeletal or visceral injury as per previously published definitions [7].

All statistical analyses were performed using IBM SPSS Statistics software (version 28). For continuous or ordinal data, the Mann–Whitney *U* test was used to compare two independent groups, whereas Fisher's exact test was used to compare two independent groups for categorical data. Data are presented as the mean (standard deviation), median (range), or number (%), as appropriate. A *p* value of <0.05 was considered as statistically significant.

A comparison between seat-belted and unbelted injured patients was performed with regard to age, sex, site of sternal fracture, presence and degree of displacement of the fracture, ECG results, GCS, and ISS. Another comparison between patients with an isolated sternal fracture and patients with other concomitant injuries was performed regarding different variables.

## 3. Results

During the study period, 5632 patients with blunt trauma were presented to our hospital. A chest CT scan was performed for 2578 (45.8%) patients. Sternal fractures were diagnosed in 63 (2.4%) patients; of these, 56 (88.8%) were men and 7 (11.1%) were women (Table 1).

The patient's median (range) age was 37 (7–91) years. MVC was the main mechanism of injury in 56 (88.8%) patients followed by falling from a height in six (9.5%) patients. In 49 patients with known seatbelt usage status, 38 (77.6%) were belted and 11 (22.4%) were unbelted.

No statistically significant differences were noted between the seat belted and unbelted patients regarding age, sex, site of sternal fracture, presence or degree of displacement of the fracture, ECG results, GCS, and ISS (Table 2).

The median mean arterial pressure of the patients at presentation to the emergency department was 101 (84–220) mmHg, and the median pulse was 83.5 (64–143) bpm.

An anteroposterior chest X-ray scan was completed before the CT scan in 15 (23.8%) patients; one of them also showed a lateral view of the chest. A fracture of the sternum was exclusively identified in the lateral view, with none of the anteroposterior chest radiographs revealing any signs of an existing sternal fracture.

The body was the most fractured region of the sternum, which was identified in 47 (74.6%) patients. No fractures involved the xiphisternum. The sternal fracture was displaced in 28 (44.4%) patients. The mean displacement of the fractures was 2 (1–10) mm (Table 1).

Twenty (31.7%) patients had an isolated sternal fracture with no other associated injuries. Six (30%) of those patients had an abnormal ECG but normal troponin levels. None of the patients with isolated sternal fractures was admitted to the ICU, and five (25%) of them were discharged home from the emergency department as they did not have any ECG changes and their cardiac enzymes were normal.

Patients with an isolated sternal fracture had statistically significant less admission to the hospital, a lower ISS, and significantly shorter hospital stay days compared with patients who had other concomitant injuries. There was no statistical difference between the two groups with regard to age, mechanism of injury, fracture displacement, GCS, ECG results, and cardiac enzyme abnormalities (Table 3).

Forty-three (68.3%) patients had other concomitant injuries in addition to the sternal fracture. The chest was the most frequently injured region (31 patients, 49.2%), followed by the spine (16 patients, 25.4%) and lower limbs (11 patients, 17.5%) (Table 4). Four patients had associated head injuries, one of whom died and two experienced long-term neurological sequelae. Rib fracture was the most common chest injury associated with sternal fracture (17 patients, 27%), followed by lung contusions (14 patients, 22.2%) (Table 5).

ECG results were available in the records of 43 patients. Sixteen (37.2%) of them were found to have an abnormal ECG; of these, only two (12.5%) had elevated troponin levels. Four patients with an abnormal ECG and normal cardiac enzyme levels had a history of cardiac disease. No correlation was detected between ECG results and troponin levels (*p*=1.00; Fisher's exact test).

Six (11.1%) of the forty-three patients with available ECG data had an elevated troponin level. These patients were also found to have elevated levels of CK and CK-MB. Two of them died (one had an abnormal ECG, and the other had no available ECG data). The available cardiac enzyme level data are presented in Table 6.

The median (range) GCS was 15 (3–15), and the median (range) ISS was 9 (4–57). The total days of hospital stay median (range) was 56 (1–750) days. Two patients had spastic quadriparesis and stayed in the hospital for a long time (205 and 750 days) in a vegetative state following a severe head injury. Seven patients (11.1%) were admitted to the ICU, all of them had other associated injuries in addition to the sternal fracture, and the median (range) ICU stay was 5.5 (1–50) days (Table 1).

In the current study, the sternal fracture in all the patients was treated nonoperatively (conservative management).

Four patients were transferred to another hospital. Three of them had spinal fractures that required fixation by a neurosurgeon, and the fourth patient showed active bleeding from a hepatic injury requiring embolization by interventional radiologists. Unfortunately, neurosurgery and interventional radiology are not currently available at our hospital.

Seven (11.1%) patients were discharged directly from the emergency department, and all of them had normal ECGs and troponin levels. Five patients had an isolated sternal fracture.

**Figure 1:**
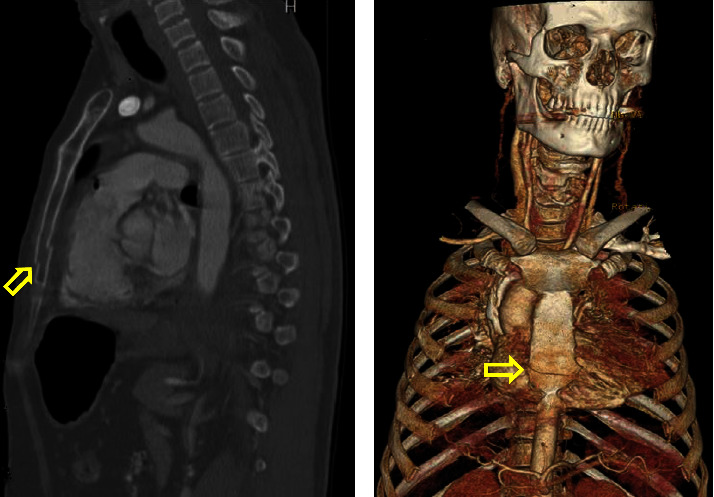
A 56-year-old male truck driver involved in a motor vehicle collision. He had multiple lower limb fractures, hemothorax, multiple fractured ribs, and hypotension. He had an abnormal ECG and an elevated troponin level at the time of his presentation to the emergency department. He was admitted to the ICU and died on day 5 after admission. Computed tomography (CT) scan showing (a) sagittal reformat with displaced fracture of the body of the lower part of the sternum (yellow arrow), which was initially missed on axial slices; (b) 3-dimensional model showing fracture of the body of the sternum (yellow arrow).

Two patients died in this study (overall mortality rate, 3.2%). One patient was a truck driver involved in a MVC. He presented with multiple lower limb fractures, multiple rib fractures, hemothorax, and hypotension. He had an abnormal ECG and high troponin levels at presentation. He was admitted to the ICU and died on day 5 after admission because of a cardiac injury (Figure 1).

The second patient was the driver of a car who fell off a bridge. He sustained a severe head injury with fractures of multiple facial bones, severe trauma requiring amputation of his left lower limb, multiple fractured ribs, and bilateral hemothorax. Immediately after reaching the hospital, the patient was unconscious and hypotensive with fixed and dilated pupils. His ECG data were not available in the records; however, his serum troponin level was high. He was admitted to the ICU and died on day 10 after admission, primarily due to a severe head injury.

## 4. Discussion

Hospital admission of patients with an isolated sternal fracture for observation is the standard of care in several countries [8]. This study shows that routine hospital admission may be unnecessary for numerous patients with isolated sternal fractures. Five (25%) patients with an isolated sternal fracture who had a normal ECG and troponin enzyme levels were discharged directly from the emergency department. A greater proportion of those patients should have been discharged because the existing guidelines suggest that the patients of isolated sternal fracture who had a normal ECG and troponin enzyme can be managed safely in an outpatient setting [9–11]. However, at our institution, the physicians may feel that it is safer to keep the patients under observation with pain control for a short period before discharge.

Similar to that in other studies, the main mechanism of injury in this study was MVC (56 patients, 88.8%) [2, 12–14]. Some studies have reported that the introduction of seatbelt legislation has resulted in an increased frequency and severity of sternal fractures [2, 15, 16]. However, this study showed that there were no statistically significant differences between restrained and unrestrained patients in terms of the site of the sternal fracture, the presence and degree of displacement of the fracture, the ECG results, and the ISS. This can be explained by the development of deceleration injuries and blunt anterior chest wall trauma in unrestrained car occupants against the driving wheel and other objects in the car, which is similar to the deceleration injury resulting from the seatbelt. The increased number of diagnosed fractures of the sternum could be associated with increased diagnostic accuracy, which is attributable to the increased use of CT for patients with trauma [6].

Lateral chest X-ray is more specific than anteroposterior chest X-ray in diagnosing sternal fractures [2]. Patients with multiple traumatic injuries are usually brought to the hospital in the supine position following the guidelines of advanced trauma life support [17]. Accordingly, a portable supine anteroposterior chest X-ray is usually used as a screening tool for chest injuries, such as hemothorax, pneumothorax, and rib fractures. The frequency of using a lateral chest X-ray view in patients suspected of sternal fractures is low. Fifteen (23.8%) patients in this study underwent a chest X-ray as a part of their initial evaluation. Only one of these patients had a lateral view during the chest X-ray, and this additional view led to the detection of a sternal fracture. Based on ATLS principles, chest X-ray is an integral part of the primary survey during the initial resuscitation phase and should be a routine examination for multiple trauma patients (17). This arrangement was not followed at our institution by most of the patients. We think that most of the emergency physicians (all are ATLS provider certificates) send the patients directly to a CT scan to save time for the patients' management if there are no serious chest injuries based on the clinical examination.

A CT scan is currently considered the gold standard in trauma settings for the diagnosis of sternal fractures. Similar to the results reported in other studies, 62 (98.4%) patients were diagnosed with a sternal fracture on a chest CT scan [3]. The sensitivity of CT scanning is superior to that of lateral chest X-rays in diagnosing sternal fractures [6]. Furthermore, CT scanning can detect associated injuries in patients with multiple traumas [3].

The depth of sternal displacement is considered a sign of an increased risk of injury to adjacent structures such as the heart and lungs [18]. Similar to that reported in other studies, this study shows that 28 (44.4%) patients had some degree of sternal fracture displacement [19]. However, no statistically significant differences were observed in sternal fracture displacement between patients with multiple injuries and those with an isolated sternal fracture. The displaced sternal fractures are considered theoretically more dangerous, although previous studies have shown no statistically significant association between displaced fractures and the severity of blunt cardiac injuries or associated lung injuries [9, 18]. Similar to the results reported in a previous study, sternal fractures occur most frequently in the body of the sternum, and no fractures have yet reported the involvement of the xiphisternum [20].

Blunt trauma resulting in cardiac contusion can cause life-threatening arrhythmias and cardiac failure. Myocardial contusions are challenging to detect and diagnose in patients with trauma as they lack specific symptoms; moreover, there are no ideal tests to detect myocardial damage [13, 21].

An ECG may be a useful test during the initial investigation to exclude the possibility of blunt cardiac injury. ECG abnormalities are often observed in patients with trauma due to several causes, such as hypoxia, anemia, and abnormal concentrations of serum electrolytes [21]; therefore, ECG alone is not informative enough and should be coupled with cardiac enzyme levels and clinical presentation of the patient [11]. Previous studies have suggested assessing the level of troponin along with an ECG to identify patients who may have sustained blunt traumatic cardiac injury [22, 23]. In our study, the ECG alone was not significantly associated with the troponin level. Sixteen (37%) patients with available ECG reports had abnormal findings. However, only two of them (12.5%) had an elevated troponin level.

Elevated cardiac troponin levels are known to be an accurate indicator for myocardial injury. A normal troponin level has been reported to be a strong indicator for the absence of cardiac injury in patients with blunt chest trauma [14, 23]. Our study showed that in all patients with elevated troponin levels (six patients), the levels of the other available cardiac enzymes were also elevated. Only two patients of the patients with an elevated troponin level also had an abnormal ECG. Similar to other studies, our study confirms that cardiac enzyme levels are superior to ECG findings in confirming cardiac injury, particularly cardiac troponin levels [2]. Prior studies have suggested that patients with an abnormal ECG or those who are clinically unstable should undergo further investigation via assessment of cardiac enzyme levels and echocardiography [12].

Cardiac contusions can be detected via localized abnormalities in heart wall movements. Echocardiography provides a direct view of real-time myocardial motion abnormalities and has been used in the detection of cardiac contusion [24]. Patients with a sternal fracture may report pain while performing transthoracic echocardiography. In such cases, transesophageal echocardiography can be performed under sedation for the conscious patient [21]. In this study, echocardiography was not routinely used in patients with suspected blunt cardiac contusion.

Combined sternal fracture and spinal injury were observed in patients with multiple traumas [25]. In this study, a spinal fracture was the second most common injury associated with a sternal fracture in 16 (25.4%) patients. A sternal fracture may lead to a spinal fracture, particularly in the thoracic spine, as both are associated with the thoracic stability mechanism [5]. Three (18.8%) patients with spinal fractures were transferred to a tertiary center for spinal surgery, whereas the other 13 (81.2%) patients with spinal fractures were managed nonoperatively at our institution. Indications for spinal fixation include unstable fractures or fractures with spinal cord injury [26].

The management of trauma patients at our institution is in accordance with ATLS guidelines. Any life-threatening conditions should be identified and immediately treated [17]. In the current study, the sternal fracture in all the patients was treated nonoperatively (conservative management).

There was no specific protocol for the nonoperative management of the sternal fracture. However, proper analgesia for pain management was the cornerstone treatment of a sternal fracture along with adequate rest and breathing exercises in the rehabilitation period. Pain management is essential to decrease the risk of respiratory complications whether using local or systemic analgesia [27].

Patients with an isolated sternal fracture who were otherwise well and in no need for further interventions were treated with oral prescription analgesia. No radiological controls were taken, and the patients were not instructed neither to follow a specific movement restriction nor a specific decubitus.

There are no consensus guidelines regarding patient selection for the surgical stabilization of a sternal fracture [9]. Indications for operative sternal fixation include unstable fractures, severe displacement or subluxation, and symptomatic malunion or nonunion [28]. Surgical fixation options include stainless steel wires, absorbable plates, nonabsorbable plates, and internal cemented screws [9]. However, in a recent study, compared with nonoperative management, operative sternal fixation had a longer hospital stay, an extended ICU stay, and longer ventilator days. Meanwhile, operative fixation and nonoperative management had a similar rate of pneumonia, adult respiratory distress syndrome, unplanned intubation, and other complications [29].

The mortality and morbidity of patients with sternal fractures are usually not associated with the sternal fracture itself but are attributed to other associated injuries [6]. Similar to that reported in other studies, the overall mortality rate in our study was 3.17% [3].

There are certain limitations to our study. It was a retrospective study with a relatively small sample size. The strength of our study was that it was conducted in a community-based hospital, which is important because 59 (93.7%) of our patients were managed in this setting. Only 4 (6.3%) patients were transferred to tertiary hospitals due to the unavailability of advanced services at our institution. Nevertheless, the findings of our study are similar to those reported by large trauma centers and the National Trauma Data Bank with 23985 cases of blunt sternal fracture [12]. Therefore, our findings can be generalized to other tertiary hospitals.

## 5. Conclusions

A fracture of the sternum is a relatively uncommon event. The incidence of sternal fractures in a community-based hospital is similar to that reported by tertiary hospitals. Sternal fractures are often associated with severe trauma and multiple concomitant injuries. A CT scan is the imaging modality of choice for the diagnosis of sternal fractures. Most sternal fractures do not require surgical intervention. Patients with normal cardiac enzyme levels and an ECG may be safely discharged without being admitted to the hospital, provided there are no other major injuries.

## Figures and Tables

**Table 1:** The frequency distribution of patients across different variables.

Variables	*n*	(%)
Gender		
Male	56	88.9
Female	7	11.1
Mechanism of injury		
Motor vehicle collision	55	87.3
Fall	6	9.5
Others	2	3.2
Seat belt in MVC		
Yes	38	77.6
No	11	22.4
Chest X-ray		
Normal	4	6.3
Sternal fracture	1	1.6
Other abnormalities	10	15.9
Not performed	48	76.2
Type of fracture		
Transverse	17	27.0
Oblique	45	71.4
Comminuted	1	1.6
Site of sternal fracture		
Manubrium	14	22.2
Manubrium and upper body	2	3.2
Upper body	28	44.4
Middle body	9	14.3
Lower body	10	15.9
Xiphisternum	0	0
Fracture displacement		
Yes	28	44.4
No	35	55.6
ICU admission		
Yes	7	11.1
No	56	88.9

**Table 2:** Comparison between belted and unbelted MVC patients with sternal fracture in terms of different variables.

Variables		Seat belt		Total	*p* value
		Yes	No		
Age	Mean (Stdev)	40.5 (13.9)	35.8 (16.8)	39.7 (14.2)	0.510^a^

Sex	Male	35 (92.1)	9 (81.8)	44 (89.9)	0.311^b^
	Female	3 (7.9)	2 (18.2)	5 (10.1)	

Site of fracture	Manubrium and upper body	29 (76.3)	7 (63.6)	36 (73.5)	0.451^b^
	Middle and lower body	9 (23.7)	4 (36.4)	13 (26.5)	

Displacement	Yes	16 (42.1)	7 (63.6)	23 (46.9)	0.306^b^
	No	22 (57.9)	4 (36.4)	26 (53.1)	

Displacement (mm)	Mean (Stdev)	2 (2.31)	1.43 (0.79)	1.83 (1.97)	0.805^b^

ECG	Normal	18 (64.3)	4 (66.7)	22 (64.7)	1.000^b^
	Abnormal	10 (35.7)	2 (33.3)	12 (35.3)	

Troponin	Normal	30 (93.8)	10 (100)	40 (95.2)	1.000^b^
	High	2 (6.3)	0 (0)	2 (4.8)	

ISS	Mean (Stdev)	10 (6.84)	14.9 (16.52)	11 (9.62)	0.650^a^

GCS	Mean (Stdev)	38 (100)	10 (90.9)	48 (98)	0.224^b^

^a^: Fisher's exact test; ^b^: Mann–Whitney test; Stdev: standard deviation.

**Table 3:** Comparison between patients with an isolated sternal fracture and those with other concomitant injuries in terms of different variables.

Variables		Isolated sternal fracture	Multiple injuries	Total	*p* value
Age	Mean (StDev)	37.55 (16.9)	40.91 (14.9)	39.84 (15.5)	0.258^b^

Sex	Male	17 (85.0)	39 (90.7)	56 (88.9)	0.669^a^
	Female	3 (15.0)	4 (9.3)	7 (11.1)	

Mechanism	MVC	16 (80.0)	41 (95.3)	57 (90.5)	0.075^a^
	Fall	4 (20.0)	2 (4.7)	6 (9.5)	

Seat belt	Yes	11 (73.3)	27 (79.4)	38 (77.6)	0.716^a^
	No	4 (26.7)	7 (20.6)	11 (22.4)	

Displacement	Yes	7 (35.0)	21 (48.8)	28 (44.4)	0.415^a^
	No	13 (65.0)	22 (51.2)	35 (55.6)	

Displacement (mm)	Mean (StDev)	1.29 (0.5)	2.19 (2.4)	1.96 (2.2)	0.678^b^

GCS	Mean (StDev)	15.00 (0.0)	14.30 (2.6)	14.52 (2.2)	0.230^b^

ISS	Mean (StDev)	4.27 (0.5)	14.98 (11.8)	12.05 (11.1)	≤0.001^b^

ECG	Normal	7 (53.8)	20 (66.7)	27 (62.8)	0.502^a^
	Abnormal	6 (46.2)	10 (33.3)	16 (37.2)	

Troponin	Normal	19 (100.0)	29 (82.9)	48 (88.9)	0.080^a^
	High	0 (0.0)	6 (17.1)	6 (11.1)	

Hospital stays (days)	Mean (StDev)	2.35 (1.9)	26.30 (117.1)	18.70 (97)	0.013^b^

Hospital admission	Yes	15 (75.0)	41 (95.3)	56 (88.9)	0.028^a^
	No	5 (25.0)	2 (4.7)	7 (11.1)	

^a^: Fisher's exact test; ^b^: Mann–Whitney test; Stdev: standard deviation. MVC: motor vehicle collisions.

**Table 4:** Concomitantly injured body regions other than the sternum.

Region	*n*	(%)^*∗*^
Chest	31	49.2
Spine	16	25.4
Lower limb	11	17.5
Upper limb	6	9.5
Head	4	6.4
Abdomen	4	6.4

^*∗*^The percentage exceeds 100% because patients may have more than one injured body region.

**Table 5:** Chest injuries in patients with sternal fractures.

Injury	*n*	(%)^*∗*^
Fractured sternum	63	100
Fractured ribs	17	27
Lung contusion	14	22.2
Pneumothorax	8	12.7
Retrosternal hematoma	8	12.7
Fractured clavicle	5	7.9
Thoracic spine injury	5	7.9
Hemothorax	3	4.8
Fractured scapula	3	4.8

^*∗*^The percentage exceeds 100% because patients may have more than one injury.

**Table 6:** ECG and cardiac enzyme levels in admitted patients with sternal fractures.

	Available results	Normal (%)	Abnormal/High (%)
ECG	43	27 (62.8%)	16 (37.2%)
Creatine kinase (CK)	36	15 (41.7%)	21 (58.3%)
Creatine kinase MB (CK-MB)	35	24 (68.6%)	11 (31.4%)
Troponin	54	48 (88.9%)	6 (11.1%)

## Data Availability

The data used to support the findings of this study are available from the corresponding author upon request.
